# A Simple Structure-Switch Aptasensor Using Label-Free Aptamer for Fluorescence Detection of Aflatoxin B1

**DOI:** 10.3390/molecules27134257

**Published:** 2022-07-01

**Authors:** Chao Wang, Hao Yu, Qiang Zhao

**Affiliations:** 1 State Key Laboratory of Environmental Chemistry and Ecotoxicology, Research Center for Eco-Environmental Sciences, Chinese Academy of Sciences, Beijing 100085, China; wangchao@lyu.edu.cn (C.W.); tina_3127@163.com (H.Y.); 2 University of Chinese Academy of Sciences, Beijing 100049, China; 3 School of Environment, Hangzhou Institute for Advanced Study, UCAS, Hangzhou 310000, China

**Keywords:** aptamer, aflatoxin B1, fluorophore, quencher, structure-switch

## Abstract

Aflatoxin B1 (AFB1) is one of the mycotoxins produced by *Aspergillus flavus* and *Aspergillus parasiticus*, and it causes contamination in foods and great risk to human health. Simple sensitive detection of AFB1 is important and demanded for food safety and quality control. Aptamers can specifically bind to targets with high affinity, showing advantages in affinity assays and biosensors. We reported an aptamer structure-switch for fluorescent detection of aflatoxin B1 (AFB1), using a label-free aptamer, a fluorescein (FAM)-labeled complementary strand (FDNA), and a quencher (BHQ1)-labeled complementary strand (QDNA). When AFB1 is absent, these three strands assemble into a duplex DNA structure through DNA hybridization, making FAM close to BHQ1, and fluorescence quenching occurs. In the presence of AFB1, the aptamer binds with AFB1, instead of hybridizing with QDNA. Thus, FAM is apart from BHQ1, and fluorescence increases with the addition of AFB1. This assay allowed detection of AFB1 with a detection limit of 61 pM AFB1 and a dynamic concentration range of 61 pM to 4 μM. This aptamer-based method enabled detection of AFB1 in complex sample matrix (e.g., beer and corn flour samples).

## 1. Introduction

Many agricultural products (e.g., maize, corn, wheat, oilseed, peanut, and several other cereals) and fruits suffer contamination of aflatoxins [1], toxic metabolites produced by *Aspergillus flavus* and *Aspergillus parasiticus*. Aflatoxins may also be found in other contaminated food products (e.g., beer, liquor, and grape wine) [2]. The ingestion of aflatoxins threatens human and animal health [3]. Aflatoxin B1 (AFB1) is the most toxic component of aflatoxins fractions [4,5]. The International Agency for Research in Cancer (IARC) has classified AFB1 as a Group 1 carcinogen [6]. Detection of AFB1 is important in food safety and quality control. The conventional AFB1 detection methods (e.g., HPLC and LC-MS) are time consuming and require sophisticated instruments, having limitations in rapid, high-throughput and on-site test [7]. Antibody based assays allow rapid detection of AFB1 [8], but antibodies are costly and vulnerable to denature [9]. Hence, it is still desirable to develop simpler methods for rapid, high-throughput, and cost-effective detection of AFB1 on site.

Aptamer is single stranded DNA or RNA that can specifically bind to a target molecule with high affinity [10]. The aptamer is a promising rival of the antibody, and shows some advantages (e.g., in vitro selection, easy chemical synthesis, small size, high thermal stability, and low cost) [11]. It has wide applications in assays and biosensors [12]. Some aptamer-based assays for AFB1 have been developed, including fluorescence, colorimetry, electrochemistry, surface plasmon resonance, etc. [13,14,15,16,17,18]. Among these assays, fluorescence methods have attracted more attention due to their simplicity and sensitivity [19]. Fluorescence aptasensors usually rely on the target-binding induced fluorescence change, and labeling of an aptamer with fluorophore/quencher is often needed, which may change the initial affinity of the aptamer and increase cost [20]. Some nanomaterials have been used for fluorescence aptasensors, benefiting from their unique quenching ability or fluorescence property [21]. However, preparation of nanomaterials is complicated, and functionalization of nanomaterials is laborious. Nutiu and Li reported a structure-switch signaling aptamer strategy [22] in which intact label-free aptamers were used as recognition units for fluorescence detection of target molecules. This strategy is general and has few restrictions on the size and secondary structure of aptamers. To date, this structure-switch signaling aptamer strategy has been successfully applied to detections of some targets, such as adenosine triphosphate (ATP), thrombin [22], and chloramphenicol [23]. 

In this study, we describe a fluorescent switch aptasensor for detection of AFB1 using a label-free aptamer, a fluorescein (FAM)-labeled complementary DNA (denoted as FDNA), and a quencher (BHQ1)-labeled complementary DNA (denoted as QDNA). In the absence of AFB1, a DNA duplex of the aptamer, FDNA, and QDNA is formed through DNA hybridization. This DNA assembly brings FAM and BHQ1 into proximity, causing fluorescence quenching. When AFB1 is present, the aptamer prefers to bind with AFB1, as the aptamer–AFB1 complex is more stable than the DNA duplex assembly. Aptamer–AFB1 binding causes release of QDNA from DNA duplex, and fluorescence intensity increases. Thus, detection of AFB1 is achieved by measuring the fluorescence change. Under optimized assay conditions, we achieved quantitative detection of AFB1 in a concentration range of 61 pM to 4 μM, with a detection limit of 61 pM AFB1. This structure-switch aptasensor allowed the detection of AFB1 in a complex sample matrix (e.g., beer and corn flour). It shows the advantages of the use of label-free aptamers and a larger enhancement of the fluorescence signal. This analytical method has potential in rapid, high-throughput, on-site and cost-effective detection of AFB1.

## 2. Results and Discussions

### 2.1. Working Principle of Fluorescent Switch Aptasensor for AFB1 Detection

**Figure 1:**
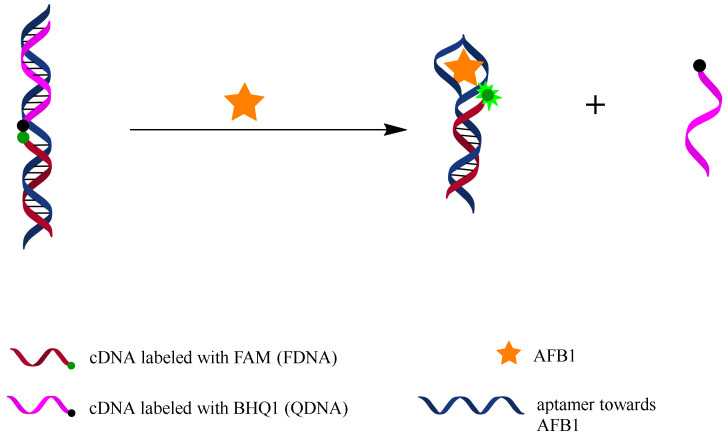
Working principle of fluorescent switch aptasensor for AFB1 detection. In the absence of AFB1, aptamer hybridizes with both FDNA and QDNA to form a tripartite DNA duplex structure in which FAM and BHQ1 are close to each other, causing fluorescence quenching. In the presence of AFB1, aptamer prefers to bind with AFB1 rather than QDNA, resulting in disassembly of the tripartite DNA duplex and fluorescence recovery. AFB1 can be detected by measuring the fluorescence change.

Figure 1 illustrates the working principle of the fluorescent switch aptasensor. FDNA is labeled with a fluorescein (FAM) at the 5’end, and QDNA is labeled with a quencher (BHQ1) at the 3’end. The unlabeled aptamer sequence contains a FDNA-hybridizing sequence and an AFB1 binding motif. In the absence of AFB1, an FDNA, QDNA, and aptamer sequence together construct a tripartite DNA duplex, which makes FAM close to BHQ1, causing fluorescence quenching. In the presence of AFB1, aptamer binds with AFB1, and QDNA is released from the DNA duplex, resulting in a fluorescence increase. AFB1 can be detected by measuring the increase in fluorescence intensity of this aptasensor. 

### 2.2. Feasibility of Fluorescent Structure-Switch Aptasensor

**Figure 2:**
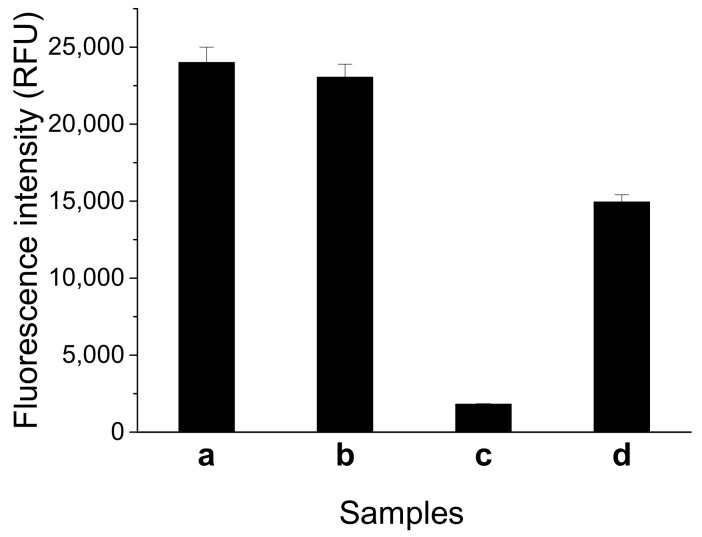
Feasibility test of the fluorescent switch aptasensor for AFB1 detection by measuring different samples. (**a**) FDNA; (**b**) FDNA+QDNA; (**c**) FDNA+QDNA+aptamer; and (**d**) FDNA + QDNA + aptamer + AFB1. Assay buffer was 10 mM Tris-HCl (pH 7.5) solution containing 50 mM MgCl_2_, 50 mM NaCl, and 0.1% Tween 20. FDNA concentration was 50 nM. Aptamer (Af27) concentration was 100 nM. QDNA (Af27-C13Q) concentration was 100 nM. AFB1 concentration was 200 nM. Incubation at 4 °C for 60 min was applied.

We tested the feasibility of the fluorescent switch aptasensor for AFB1 detection. As Figure 2 shows, the sample containing FDNA and QDNA displayed high fluorescence intensity similar to that of the sample containing only FDNA. This result means FDNA fluorescence cannot be quenched by QDNA in the absence of aptamer. After the addition of aptamer, a significant decrease in fluorescence intensity was observed. This result shows the tripartite DNA duplex was successfully constructed, and FDNA fluorescence was quenched. Fluorescence obviously increased upon addition of AFB1 (200 nM) into the solution containing FDNA, QDNA, and the aptamer. Thus, the fluorescent switch aptasensor is feasible for AFB1 detection.

### 2.3. Optimization of Experimental Conditions

To achieve better assay performances, we optimized some important experimental conditions including sequences of aptamer and QDNA, concentration of FDNA, concentration ratio between FDNA, aptamer and QDNA (*C*_FDNA_:*C*_Aptamer_:*C*_QDNA_), MgCl_2_ concentration, NaCl concentration, incubation temperature, and incubation time. In the following discussions, F_0_, F_blank_, and F_AFB1_ represent the fluorescence intensity of sample containing only FDNA; the fluorescence intensity of sample containing FDNA, QDNA, and aptamer; and the fluorescence intensity of sample containing FDNA, QDNA, aptamer, and AFB1, respectively. Quenching efficiency was calculated by (1 − (F_blank_/F_0_)) × 100%.

**Figure 3:**
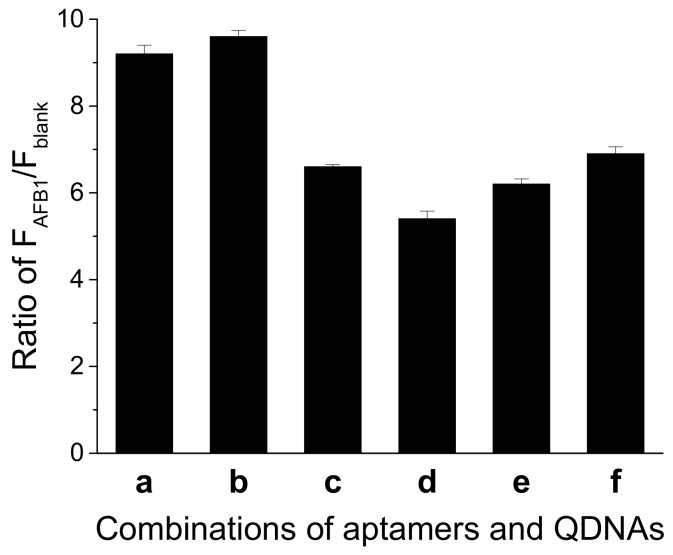
Effects of different combinations of aptamer and QDNA on the ratio of F_AFB1_/F_blank_. (**a**) Af27 + Af27 − C12Q; (**b**) Af27 + Af27 − C13Q; (**c**) Af27 + Af27 − C14Q; (**d**) Af29 + Af29 − C13Q; (**e**) Af29 + Af29 − C14Q; and (**f**) Af29 + Af29 − C15Q. Assay buffer was 10 mM Tris-HCl (pH 7.5) solution containing 50 mM MgCl_2_, 50 mM NaCl, and 0.1% Tween 20. FDNA concentration was 50 nM. Aptamers (Af27 or Af29) concentrations were 100 nM. QDNAs concentrations were 100 nM. AFB1 concentration was 200 nM. Incubation at 4 °C for 60 min was applied.

A suitable DNA duplex structure is essential for the structure-switch aptasensor to generate signal response. An unstable structure gives rise to a high background, while a too stable structure might lead to weak signal response to AFB1, causing a reduction in sensitivity. We explored different combinations of the aptamer and QDNA. As shown in Figure 3, when Af27 and Af27-C13Q were employed, a larger ratio of F_AFB1_ to F_blank_ (F_AFB1_/F_blank_) was obtained. Therefore, aptamer Af27 and QDNA Af27-C13Q were applied in the further experiments.

**Figure 4:**
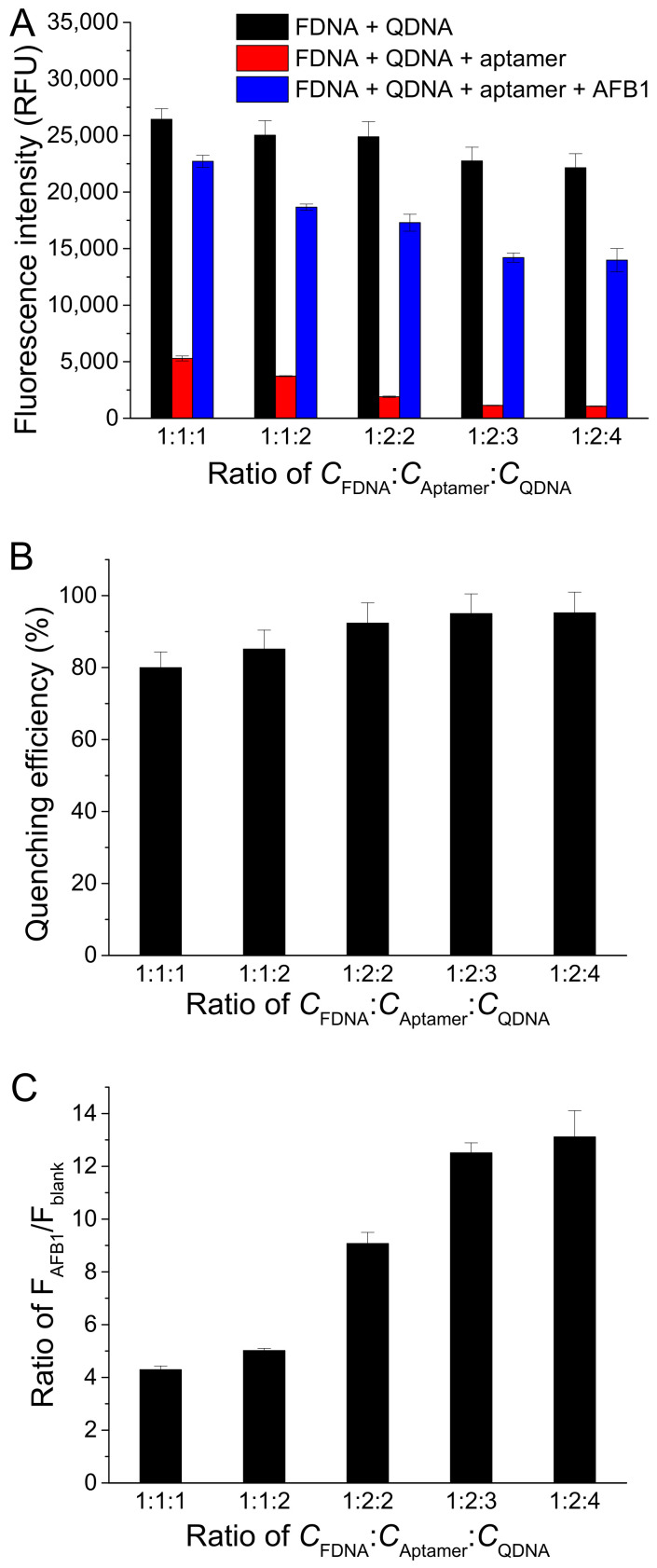
Effects of different concentration ratios between FDNA, aptamer, and QDNA (*C*_FDNA_:*C*_Aptamer_:*C*_QDNA_) on (**A**) fluorescence intensity of samples, (**B**) quenching efficiency, and (**C**) ratio of F_AFB1_/F_blank_. Assay buffer was 10 mM Tris-HCl (pH 7.5) solution containing 50 mM MgCl_2_, 50 mM NaCl, and 0.1% Tween 20. FDNA concentration was fixed at 50 nM. AFB1 concentration was 200 nM. Af27 and Af27-C13Q were used as aptamer and QDNA, respectively. Incubation at 4 °C for 60 min was applied.

To investigate the effect of the concentration ratio between FDNA, aptamer, and QDNA (*C*_FDNA_:*C*_Aptamer_:*C*_QDNA_), we fixed FDNA concentration at 50 nM and changed the aptamer concentration and QDNA concentration. As displayed in Figure 4A, F_blank_ decreased with the increase in concentrations of aptamer and QDNA, meaning a lower background. However, F_AFB1_ also decreased with the increase in aptamer and QDNA. Finally, *C*_FDNA_:*C*_Aptamer_:*C*_QDNA_ was chosen to be 1:2:3, respectively, because the quenching efficiency and the ratio of F_AFB1_/F_blank_ approached to larger values at this condition (Figure 4B,C).

Then, the FDNA concentration was optimized under the condition that *C*_FDNA_:*C*_Aptamer_:*C*_QDNA_ was fixed at 1:2:3, respectively. With an increase in FDNA concentration, fluorescence intensities F_0_ and F_AFB1_ increased (Figure S1A), while the quenching efficiency had slight change (Figure S1B). The ratio of F_AFB1_/F_blank_ reached a maximum value when 50 nM FDNA was used (Figure S1C). Therefore, 50 nM FDNA was chosen in the further experiments.

We investigated the effect of MgCl_2_ concentration on detection of AFB1 (Figure S2). Upon addition of MgCl_2_, the quenching efficiency and the ratio of F_AFB1_/F_blank_ all increased, which can be explained due to the fact that Mg^2+^ promotes both DNA hybridization and aptamer–AFB1 binding [16,17]. Finally, 50 mM MgCl_2_ was chosen because F_AFB1_/F_blank_ reached the maximum value at this condition. Only a slight effect of NaCl concentration on fluorescence intensity was observed (Figure S3) in the presence of 50 mM MgCl_2_, consistent with previous study [16,17]. Finally, we chose to add 50 mM NaCl in assay buffer.

Incubation temperature has important influence on performance of this aptasensor. As Figure S4 shows, when the incubation temperature was set at 25 °C or 37 °C, the presence of AFB1 did not cause a significant change in fluorescence. This result can be attributed to the fact that, at these temperatures, the aptamer has low affinity [15], and AFB1 binding cannot effectively replace QDNA. In contrast, a large signal change caused by AFB1 was achieved by employing incubation at 4 °C, because the aptamer has enhanced affinity at 4 °C [15]. Therefore, incubation at 4 °C was chosen. Furthermore, incubation time was also optimized (Figure S5), and incubation for 60 min was applied to find a larger value of the ratio of F_AFB1_/F_blank_.

### 2.4. Detection of AFB1

**Figure 5:**
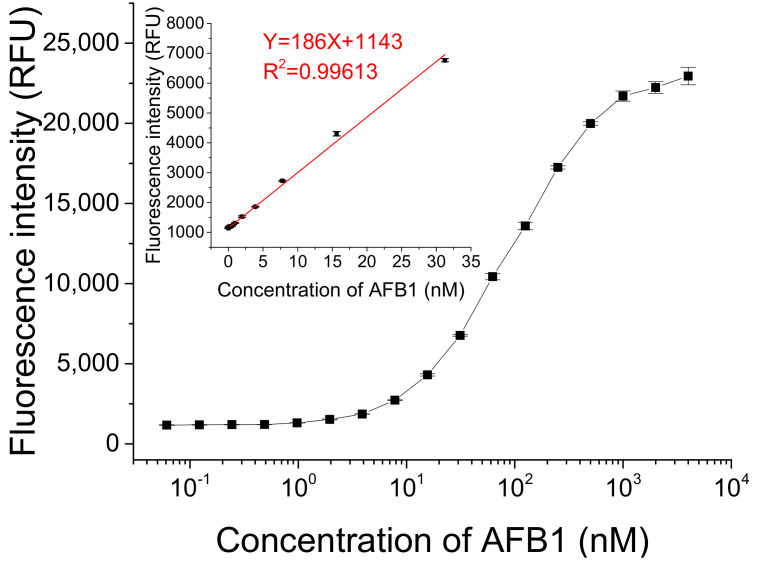
Detection of different concentrations of AFB1 using this fluorescent switch aptasensor. Assay buffer contained 10 mM Tris-HCl (pH 7.5) solution, 50 mM MgCl_2_, 50 mM NaCl, and 0.1% Tween20. Af27 and Af27-C13Q were used as aptamer and QDNA, respectively. The ratio of *C*_FDNA_:*C*_Aptamer_:*C*_QDNA_ was 50 nM:100 nM:150 nM. Incubation at 4 °C for 60 min was applied.

We detected different concentrations of AFB1 under optimized conditions. As Figure 5 shows, the fluorescence intensity gradually increased with the increasing concentration of AFB1 in a dynamic concentration range from 0 to 4 μM. A linear relationship between fluorescence intensity and AFB1 concentration ranging from 0.2 to 31.2 nM was obtained (Y = 186X + 1143 (R^2^ = 0.99613), where Y is the fluorescence intensity and X is the concentration of AFB1). The lowest concentration of AFB1 that could be detected was 61 pM, based on three times the standard deviation of the blank sample, which is lower than or comparable to the detection limits of some previously reported methods (listed in Table S1 in ESM) [13,21,24,25,26,27,28,29,30,31,32,33].

### 2.5. Selectivity Test and Detection in Complex Sample Matrix

To assess the selectivity of the structure-switch aptasensor towards AFB1, we detected AFB1 (200 nM) along with a few non-target mycotoxins including OTA, OTB, FB1, FB2, and ZAE. As shown in Figure S6, non-target mycotoxins (1 μM) gave fluorescence intensity similar to the blank sample, while AFB1 showed higher fluorescence intensity. These results demonstrate the assay was selective, and co-existences of the tested non-target mycotoxins did not cause interference to the AFB1 detection.

To evaluate the performance of this structure-switch aptasensor in a complex sample matrix, we detected different concentrations of AFB1 spiked in 20-fold diluted beer and 20-fold diluted corn flour extraction. As shown in Figure S7, in both the diluted beer sample and diluted corn flour extraction sample, the fluorescence intensity increased with the increasing spiked amount of AFB1. Detection performances in 20-fold diluted beer and 20-fold diluted corn flour extraction were comparable to that in pure assay buffer. These results demonstrate the potential application of the developed structure-switch aptasensor in real samples analysis.

## 3. Materials and Methods

### 3.1. Chemicals and Reagents

All DNA strands listed in Table 1 were synthesized and purified by Sangon Biotech Inc. (Shanghai, China). Aflatoxin B1 (AFB1), ochratoxin A (OTA), ochratoxin B (OTB), fumonisin B1 (FB1), fumonisin B2 (FB2), and zearalenone (ZAE) were purchased from Pribolab (Qingdao, China). Beer and corn flour were bought from a local supermarket. Assay buffer was 10 mM Tris-HCl (pH 7.5) solution containing 50 mM NaCl, 50 mM MgCl_2_, and 0.1% Tween20. Black 96-wells microplates were purchased from Thermo Fisher Scientific Inc. (Waltham, USA). All reagents used for experiments were analytical reagents. Solutions were prepared with ultrapure water (>18.2 MΩ·cm) from a Purelab Ultra Genetic Elga Labwater system (High Wycombe, UK).

### 3.2. Detection of AFB1

To detect AFB1, we mixed FDNA, QDNA, an aptamer, and AFB1 in the assay buffer. The final concentrations of FDNA, the aptamer, and QDNA were 50 nM, 100 nM, and 150 nM, respectively. After incubation for 60 min at 4 °C, 100 μL of the reaction mixture solution was transferred into a microplate well. Then, the fluorescence intensity was immediately measured by using a microplate reader (BioTek Synergy H1, Winooski, VT, USA), with an excitation wavelength of 485 nm and emission wavelength of 525 nm. Three repeated measurements for duplicate samples were carried out, and the average data were used.

### 3.3. Selectivity Test

To assess the selectivity of this structure-switch aptasensor towards AFB1, some non-target mycotoxins (OTA, OTB, FB1, FB2, and ZAE) were tested. The AFB1 concentration was 200 nM, and concentrations of the non-target mycotoxins were 1 μM. The assay procedures for non-target mycotoxins were the same as that for AFB1 detection.

### 3.4. Detection of AFB1 in Complex Sample Matrix

Beer was ultrasonicated to degas, and then filtered through a syringe filter (0.22 μm) before dilution with the assay buffer. Finally, different concentrations of AFB1 spiked in 20-fold diluted beer were detected using this structure-switch aptasensor following the same assay procedures. The corn flour extraction was prepared as follows: 3 mL of methanol/water (70:30, *v*/*v*) solution was added into corn flour (1 g), followed by a vortex for 20 min. After standing for 10 min, the supernatant was filtered through a syringe filter (0.22 μm) before dilution with the assay buffer. AFB1 concentrations spiked in 20-fold diluted extraction solution of corn samples were also detected by using the same assay procedures. 

## 4. Conclusions

In summary, we reported a simple fluorescent switch aptasensor for AFB1 detection using FDNA with FAM label, QDNA with BHQ1 label, and an unlabeled aptamer. In the absence of AFB1, the three DNA sequences assembled to DNA duplex, showing low fluorescence due to fluorescence quenching. The addition of AFB1 caused dissociation of the DNA duplex consisting of FDNA, QDNA, and the unlabeled aptamer, resulting in a fluorescence increase. This aptasensor allowed the sensitive detection of AFB1, and a detection limit reached 61 pM. This aptasensor was selective and allowed to detect AFB1 in diluted beer and corn flour extraction. This method shows the advantages of a larger enhancement of fluorescence signal and use of an unlabeled aptamer. This sensor is promising in rapid, high-throughput, on-site, and cost-effective detection of AFB1. 

## Figures and Tables

**Table 1:** Sequences of different DNA strands.

Name	Function	Sequence (5’ to 3’)
FDNA	FDNA	FAM-TCACAGATGAGT
Af27	aptamer	ACTCATCTGTGATCACGTGTTGTCTCTCTGTGTCTCGTG
Af29	aptamer	ACTCATCTGTGATGCACGTGTTGTCTCTCTGTGTCTCGTGC
Af27-C12Q	QDNA	GACAACACGTGA-BHQ1
Af27-C13Q	QDNA	AGACAACACGTGA-BHQ1
Af27-C14Q	QDNA	GAGACAACACGTGA-BHQ1
Af29-C13Q	QDNA	GACAACACGTGCA-BHQ1
Af29-C14Q	QDNA	AGACAACACGTGCA-BHQ1
Af29-C15Q	QDNA	GAGACAACACGTGCA-BHQ1

The underlined section of aptamers (Af27 and Af29) are the sequence binding with AFB1.

## Supplementary Materials

The following supporting information can be downloaded at: https://www.mdpi.com/article/10.3390/molecules27134257/s1, Table S1. Comparison of some aptamer-based fluorescence assays for AFB1; Figure S1. Effects of different FDNA concentrations on (A) fluorescence intensity of samples, (B) quenching efficiency, and (C) ratio of F_AFB1_/F_blank_; Figure S2. Effects of MgCl_2_ concentrations in assay buffer on (A) fluorescence intensity of samples, (B) quenching efficiency, and (C) ratio of F_AFB1_/F_blank_; Figure S3. Effects of different NaCl concentrations in assay buffer on (A) fluorescence intensity of samples, (B) quenching efficiency, and (C) ratio of F_AFB1_/F_blank_; Figure S4. Effects of different incubation temperatures on (A) fluorescence intensity of samples, (B) quenching efficiency, and (C) ratio of F_AFB1_/F_blank_; Figure S5. Effects of incubation time on (A) fluorescence intensity of samples, (B) quenching efficiency, and (C) ratio of F_AFB1_/F_blank_; Figure S6. Selectivity of the fluorescent switch aptasensor towards AFB1; Figure S7. Detections of AFB1 spiked in 20-fold diluted beer and 20-fold diluted corn flour extraction.
