# Supplementary material for: A Simple Structure-Switch Aptasensor Using Label-Free Aptamer for Fluorescence Detection of Aflatoxin B1
Source: Molecules. 2022 Jul 1;27(13):4257. doi: 10.3390/molecules27134257 (PMC9268478; doi:10.3390/molecules27134257)
Supplement: Supplementary file 1 [file molecules-27-04257-s001.zip › molecules-1769487-supplementary.pdf]

molecules

MDPI

Electronic Supplementary Material

A simple structure-switch aptasensor using label-free aptamer for fluorescence detection of aflatoxin B1

Chao Wang [1,2], Hao Yu [1,2] and Qiang Zhao [1,2,3,*]

[1] State Key Laboratory of Environmental Chemistry and Ecotoxicology, Research Center for Eco-Environmental Sciences, Chinese Academy of Sciences, Beijing 100085, China; wswcwazn@qq.com (C.W.), tina_3127@163.com (H.Y.)
[2] University of Chinese Academy of Sciences, Beijing 100049, China
[3] School of Environment, Hangzhou Institute for Advanced Study, UCAS, Hangzhou 310000, China
* Correspondence: qiangzhao@rcees.ac.cn

Table S1. Comparison of some aptamer-based fluorescence assays for AFB1.

Strategies	Label Types of Aptamer	Dynamic Detection Range	LOD	Ref.
Assay using fluorescein-labeled aptamer and quencher labeled complementary strand	Single-label	16-320 nM	5.1 nM	[13]
Fluorescent aptasensor using AIEgens and graphene oxide	Label-free	0.8-10 nM	0.8 nM	[21]
A simple fluorescence aptamer molecular beacon	Dual-label	3.9 nM-4 μM	3.9 nM	[24]
FRET-based aptasensor	Single-label	10-400 nM	3.4 nM	[25]
Aptamer with dual-terminal proximity structures	Dual-label	3.2-3200 nM	2.9 nM	[26]
Assay using a quencher system composed of quantum dots and graphene oxide	Single-label	3.2 nM-320 μM	1.0 nM	[27]
Aptamer assay using aggregation-induced emission dyes	Single-label	1.0 nM-16 μM	1.0 nM	[28]
A TAMRA quenching-based aptasensing platform	Single-label	0.8-102.4 nM	0.6 nM	[29]
A thioflavin T-based aptasensor	Label-free	0.6-640 nM	0.6 nM	[30]
Assay using fluorescein-labeled aptamer and BHQ1-labeled complementary DNA	Single-label	0.2 to 500 nM	0.2 nM	[31]
Aptasensor based on the catalytic effect of aptamer/G-quadruplex DNAzyme probe	Label-free	1.6 nM-48 nM	64 pM	[32]
A structure-switching signaling aptamer	Label-free	60 pM-4 μM	61 pM	this work
Aptasensor using DNA-guided assembly of fluorescent probe and TdT-assisted DNA polymerization	Single-label	50 pM-50 nM	10 pM	[33]

TAMRA: tetramethyl-6-carboxyrhodamine; BHQ1: black hole quencher 1

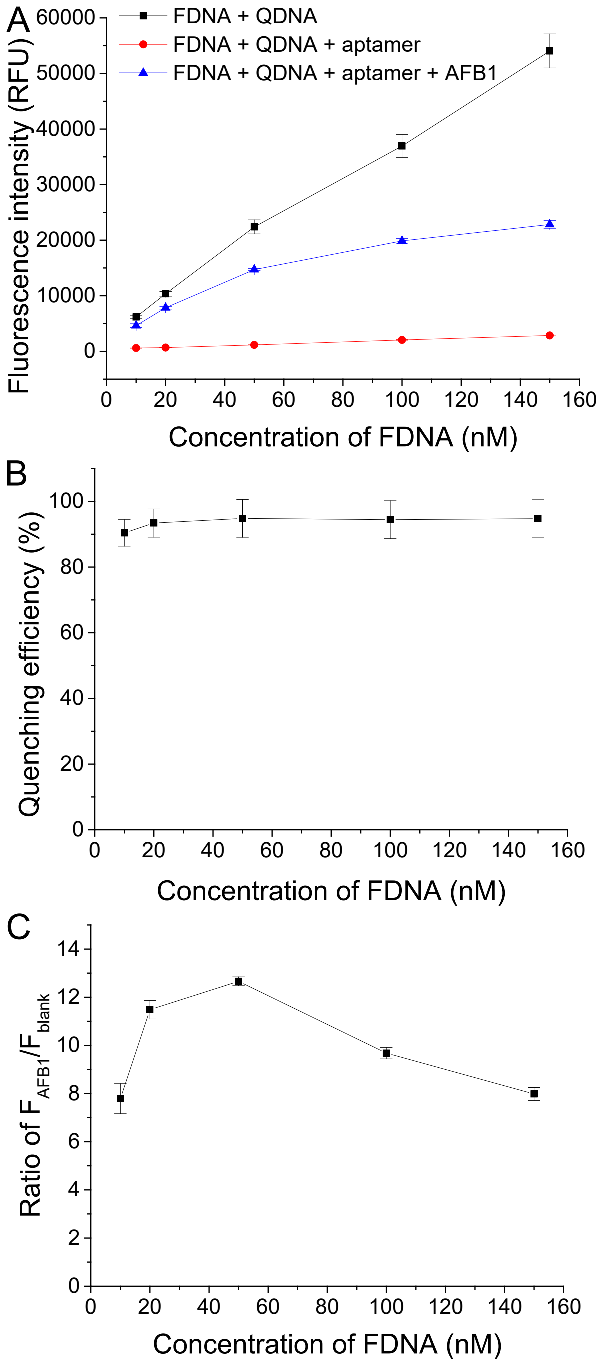

Figure. S1. Effects of different FDNA concentrations on (**A**) fluorescence intensity of samples, (**B**) quenching efficiency, and (**C**) ratio of F_{AFB1}/F_{blank}. Assay buffer was 10 mM Tris-HCl (pH 7.5) containing 50 mM $MgCl_2$, 50 mM NaCl and 0.1% Tween 20. Af27 and Af27-C13Q were used as Aptamer and QDNA, respectively. The ratio of C_{FDNA}: $C_{Aptamer}$: C_{QDNA} was fixed at 1: 2: 3. AFB1 concentration was 200 nM. Incubation at 4 ^{o}C for 60 min was applied.

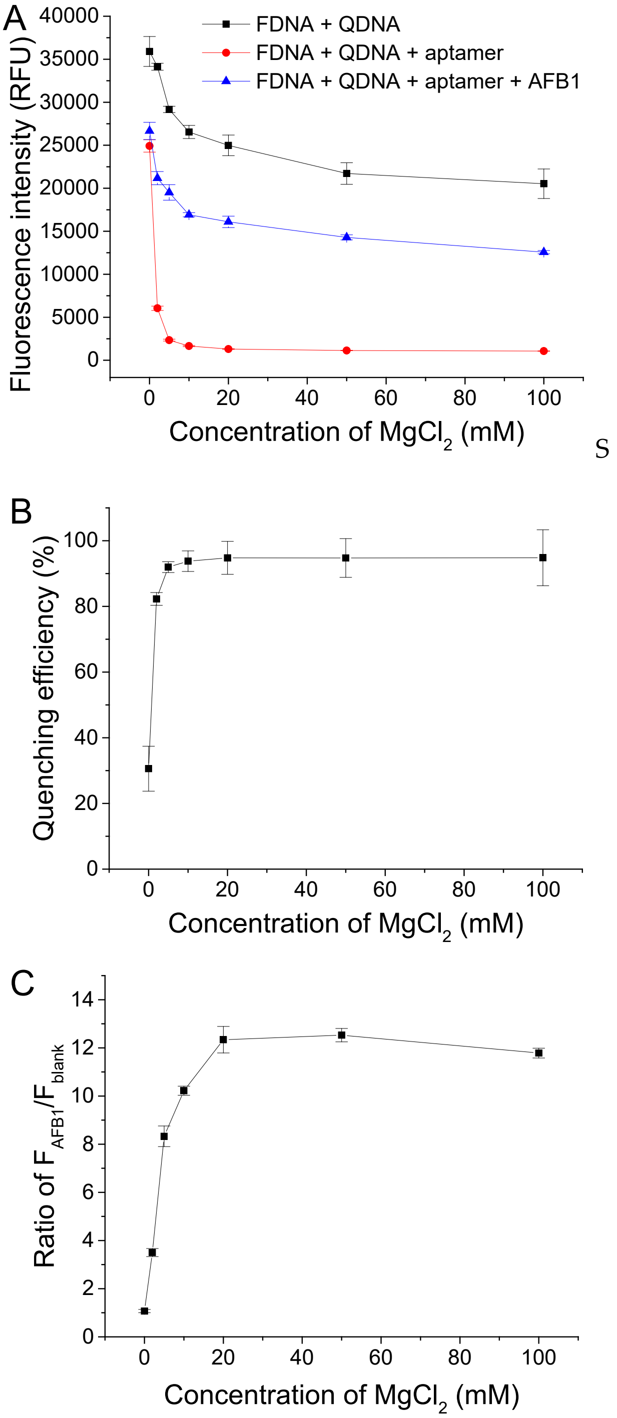

Figure. S2. Effects of $MgCl_2$ concentrations in assay buffer on (A) fluorescence intensity of samples, (B) quenching efficiency, and (C) ratio of F_{AFB1}/F_{blank}. Assay buffer was 10 mM Tris-HCl (pH 7.5) containing 50 mM NaCl, 0.1% Tween 20 and different concentrations of $MgCl_2$. Af27 and Af27-C13Q were used as aptamer and QDNA, respectively. The ratio of C_{FDNA}: $C_{Aptamer}$: C_{QDNA} was 50 nM: 100 nM: 150 nM. AFB1 concentration was 200 nM. Incubation at 4 °C for 60 min was applied.

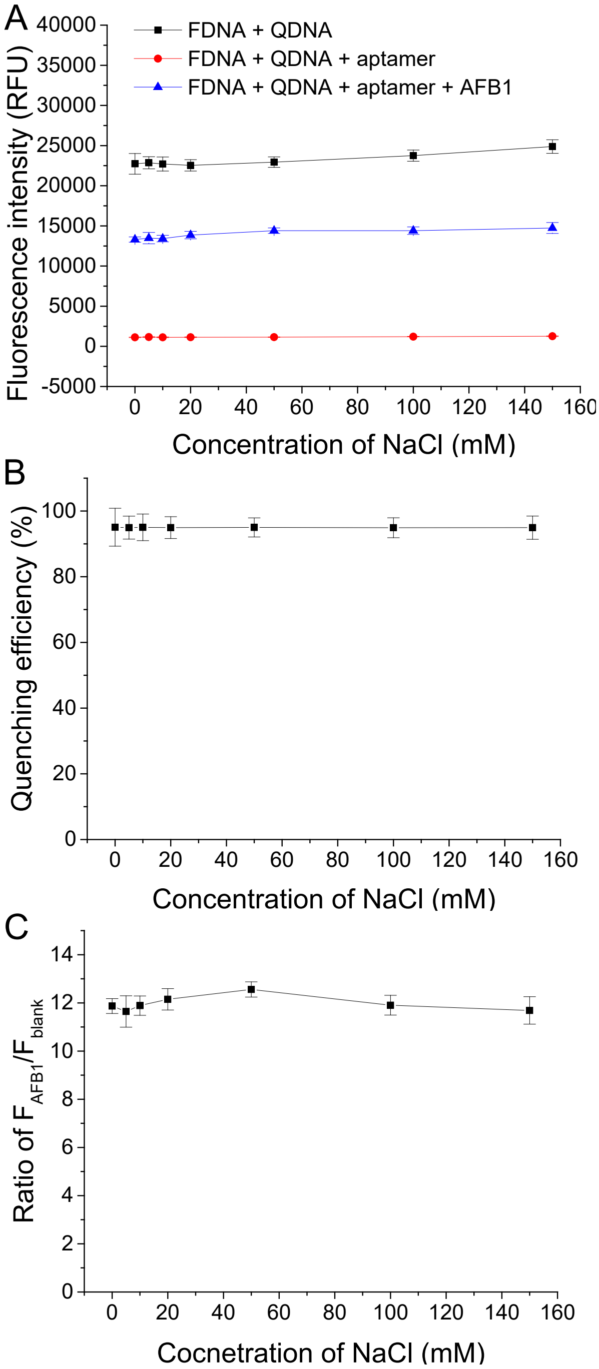

Figure. S3. Effects of different NaCl concentrations in assay buffer on (**A**) fluorescence intensity of samples, (**B**) quenching efficiency, and (**C**) ratio of F_{AFB1}/F_{blank}. Assay buffer was 10 mM Tris-HCl (pH 7.5) containing 50 mM $MgCl_2$, 0.1% Tween 20 and different concentrations of NaCl. Af27 and Af27-C13Q were used as aptamer and QDNA, respectively. The ratio of C_{FDNA}: $C_{Aptamer}$: C_{QDNA} was 50 nM: 100 nM: 150 nM. AFB1 concentration was 200 nM. Incubation at 4 °C for 60 min was applied.

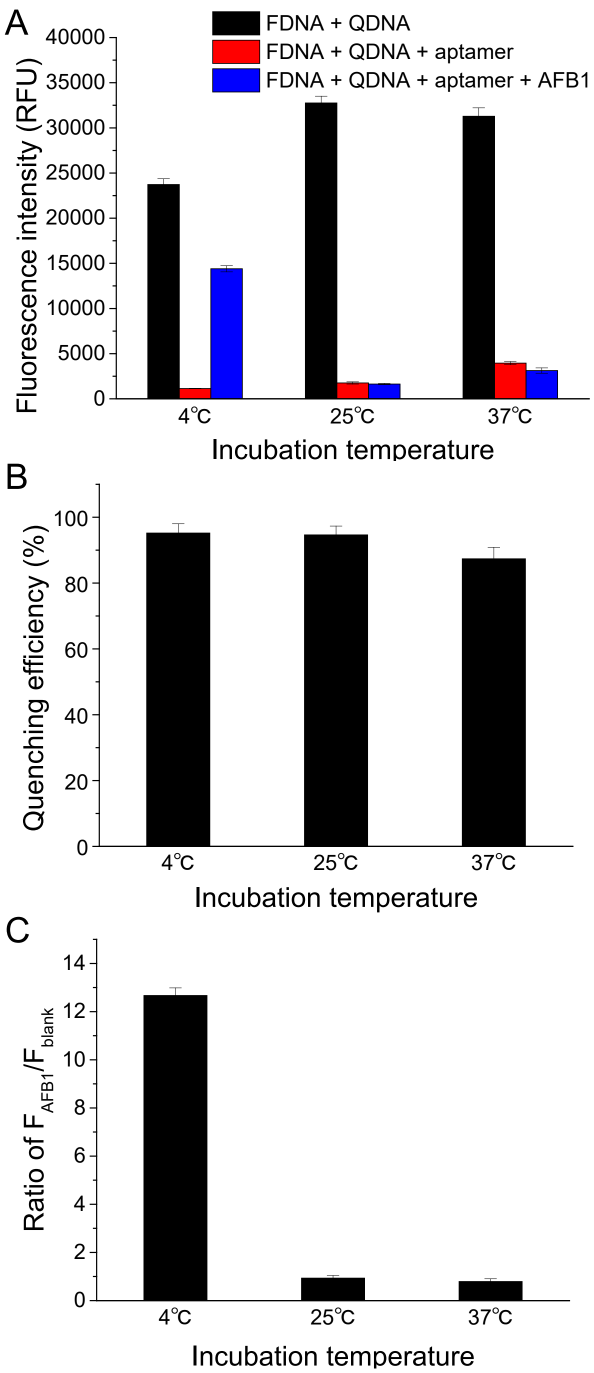

Figure. S4. Effects of different incubation temperatures on (**A**) fluorescence intensity of samples, (**B**) quenching efficiency, and (**C**) ratio of F_{AFB1}/F_{blank}. Assay buffer was 10 mM Tris-HCl (pH 7.5) containing 50 mM $MgCl_2$, 50 mM NaCl and 0.1 % Tween 20. Af27 and Af27-C13Q were used as Aptamer and QDNA, respectively. The ratio of C_{FDNA}: $C_{Aptamer}$: C_{QDNA} was 50 nM: 100 nM: 150 nM. AFB1 concentration was 200 nM. Incubations for 60 min under different temperatures were applied.

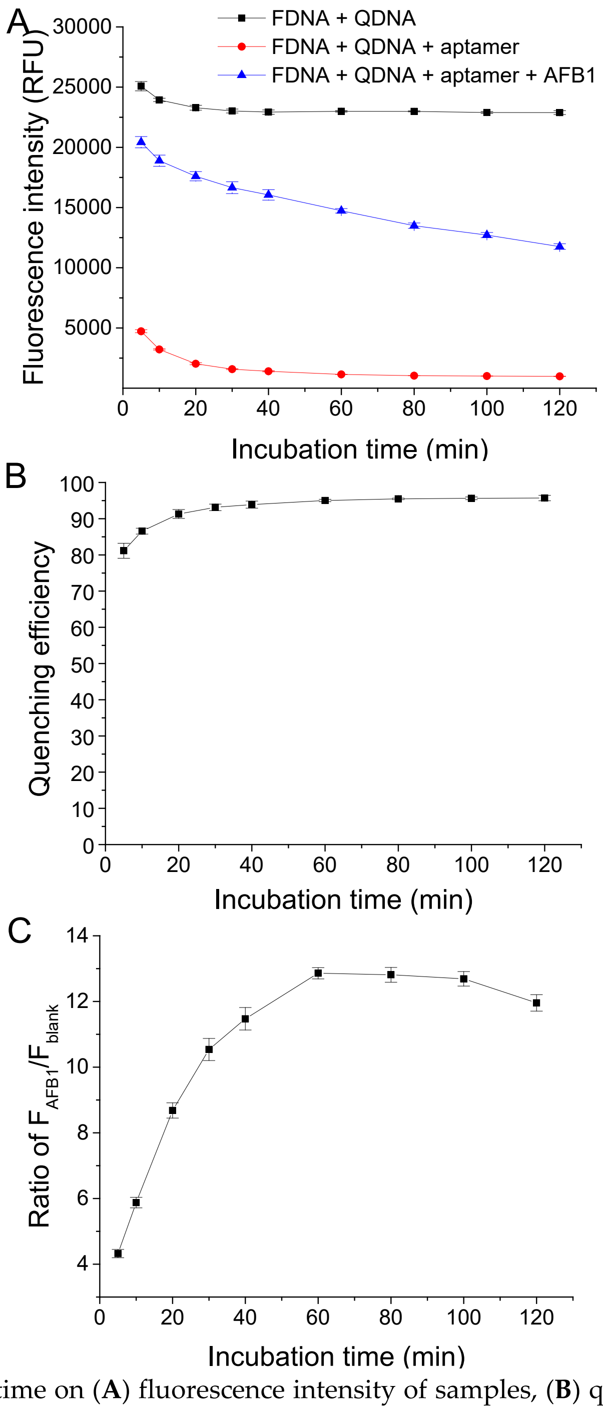

Figure. S5. Effects of incubation time on (**A**) fluorescence intensity of samples, (**B**) quenching efficiency, and (**C**) ratio of F_{AFB1}/F_{blank}. Assay buffer was 10 mM Tris-HCl (pH 7.5) containing 50 mM $MgCl_2$, 50 mM NaCl and 0.1% Tween 20. Af27 and Af27-C13Q were used as aptamer and QDNA, respectively. The ratio of C_{FDNA}: $C_{Aptamer}$: C_{QDNA} was 50 nM: 100 nM: 150 nM. AFB1 concentration was 200 nM. Incubations at 4 ^{o}C for different times were applied.

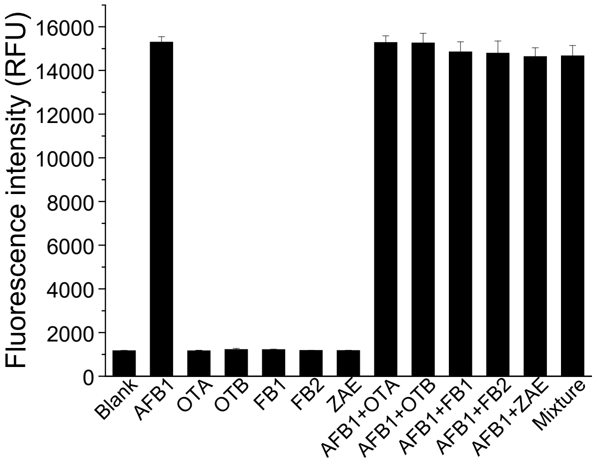

Figure. S6. Selectivity of the fluorescent switch aptasensor towards AFB1. Non-target mycotoxins including OTA, OTB, FB1, FB2, ZAE were tested. The concentration of AFB1 was 200 nM, and concentrations of non-target mycotoxins were all at 1 μM. The mixture contained AFB1, OTA, OTB, FB1, FB2, and ZAE.

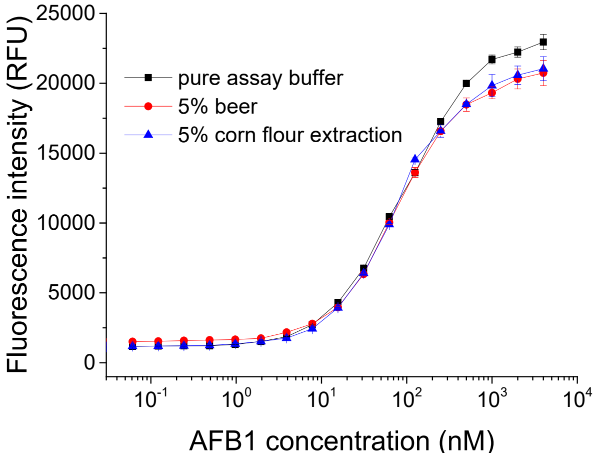

Figure. S7. Detection of different concentrations of AFB1 spiked in 20-fold diluted beer and 20-fold diluted corn flour extraction, respectively. The prepared beer and corn flour extraction samples were diluted with assay buffer (10 mM Tris-HCl (pH 7.5), 50 mM $MgCl_2$, 50 mM NaCl and 0.1% Tween 20). Af27 and Af27-C13Q were used as aptamer and QDNA, respectively. The ratio of C_{FDNA}: $C_{Aptamer}$: C_{QDNA} was 50 nM: 100 nM: 150 nM. Incubation at 4 °C for 60 min was applied.
